# A Digital Signal Processor Based Acoustic Sensor for Outdoor Noise Monitoring in Smart Cities

**DOI:** 10.3390/s20030605

**Published:** 2020-01-22

**Authors:** Juan Manuel López, Jesús Alonso, César Asensio, Ignacio Pavón, Luis Gascó, Guillermo de Arcas

**Affiliations:** Grupo de Investigación en Instrumentación y Acústica Aplicada, Universidad Politécnica de Madrid, 28040 Madrid, Spain; jalonsof@i2a2.upm.es (J.A.); casensio@i2a2.upm.es (C.A.); ignacio.pavon@upm.es (I.P.); luis.gasco@i2a2.upm.es (L.G.)

**Keywords:** outdoors noise, sound level meter, digital signal processing, multirate filters

## Abstract

Presently, large cities have significant problems with noise pollution due to human activity. Transportation, economic activities, and leisure activities have an important impact on noise pollution. Acoustic noise monitoring must be done with equipment of high quality. Thus, long-term noise monitoring is a high-cost activity for administrations. For this reason, new alternative technological solutions are being used to reduce the costs of measurement instruments. This article presents a design for a versatile electronic device to measure outdoor noise. This device has been designed according to the technical standards for this type of instrument, which impose strict requirements on both the design and the quality of the device’s measurements. This instrument has been designed under the original equipment manufacturer (OEM) concept, so the microphone–electronics set can be used as a sensor that can be connected to any microprocessor-based device, and therefore can be easily attached to a monitoring network. To validate the instrument’s design, the device has been tested following the regulations of the calibration laboratories for sound level meters (SLM). These tests allowed us to evaluate the behavior of the electronics and the microphone, obtaining different results for these two elements. The results show that the electronics and algorithms implemented fully fit within the requirements of type 1 noise measurement instruments. However, the use of an electret microphone reduces the technical features of the designed instrument, which can only fully fit the requirements of type 2 noise measurement instruments. This situation shows that the microphone is a key element in this kind of instrument and an important element in the overall price. To test the instrument’s quality and show how it can be used for monitoring noise in smart wireless acoustic sensor networks, the designed equipment was connected to a commercial microprocessor board and inserted into the infrastructure of an existing outdoor monitoring network. This allowed us to deploy a low-cost sub-network in the city of Málaga (Spain) to analyze the noise of conflict areas due to high levels of leisure noise. The results obtained with this equipment are also shown. It has been verified that this equipment meets the similar requirements to those obtained for type 2 instruments for measuring outdoor noise. The designed equipment is a two-channel instrument, that simultaneously measures, in real time, 86 sound noise parameters for each channel, such as the equivalent continuous sound level (Leq) (with Z, C, and A frequency weighting), the peak level (with Z, C, and A frequency weighting), the maximum and minimum levels (with Z, C, and A frequency weighting), and the impulse, fast, and slow time weighting; seven percentiles (1%, 5%, 10%, 50%, 90%, 95%, and 99%); as well as continuous equivalent sound pressure levels in the one-third octave and octave frequency bands.

## 1. Introduction

According to the United Nations, 55% of the world’s population currently resides in urban areas, with this percentage projected to reach 66% by 2050 [1]. This rapid urban growth has caused environmental impacts, including environmental noise exposure to citizens. Noise pollution is one of the most important environmental health concerns around world. Environmental noise is produced by a variety of sources and is widely present in urban environments. Among the adverse effects produced by environmental noise exposure are those that threaten the well-being of human populations, deteriorate health, and decrease the ability of children to learn properly at school, leading to high economic costs for society [2].

In 2018, the World Health Organization (WHO) Regional Office for Europe published Environmental Noise Guidelines for the European Region. Compared to previous WHO guidelines on noise, there are some significant developments in the new version, among which the following should be highlighted: the inclusion of leisure noise in addition to noise from transportation (aircraft, rail and road traffic), and the use of long-term average noise exposure indicators to better predict adverse health outcomes compared to short-term noise exposure measures [3,4]. Both leisure noise and long-term average noise exposure indicators are issues to be considered in the management of noise in urban environments.

The Environmental Noise Directive (END) provides mechanisms for annoyance and sleep disturbance assessment, which if exceeded require action plans to be drawn that are designed to reduce exposure and protect areas not yet polluted by noise [5]. One of the most important evaluation mechanisms of the END is strategic noise mapping. At present, only industrial noise sources and noise from means of transport are taken into account (roads, railways, and airports) for strategic noise mapping. Meanwhile, there are many other sources of noise within urban environments not covered by strategic noise maps, such as citizen behavior, festive and cultural events, public works, urban maintenance and cleaning, and leisure noise, including night-life activities.

In order to carry out a comprehensive assessment of all the noise sources present in an area, one of the options that many cities usually use is environmental noise monitoring networks [6]. Environmental noise monitoring systems consist of a network of discrete sensor stations, usually integrated with an averaging sound level meter using an outdoor microphone. One of the main advantages of environmental noise monitoring systems lies in their ability to use the required time evolution data. On the other hand, the main drawback of environmental noise monitoring systems is that the discrete number of points implies weaknesses in the representativeness of spatial data. Different approaches have been proposed in recent times to solve the problems related to spatial representativeness. Examples of this are proposals to perform environmental noise measurements based on smart phones [7,8,9] and mobile monitoring networks using means of transport [10,11]. As a general rule, the lower the cost of the sensor, the more sensors can be used and the more spatially accurate the data will be.

Traditionally, professional systems used for noise measurement are designed to comply with very high-quality measurement requirements and are manufactured under strict international standards, such as IEC 61672 and IEC 61260 [12,13,14,15,16,17]. This situation makes this kind of equipment unsuitable for creating wide grids of measurement points in smart cities due to its high cost, large size, and other factors.

Recent technological developments related to the availability of cheaper and smaller equipment and innovations in communication networks and acoustic signal processing have led to the emergence of low-cost environmental noise sensor networks [18]. In recent years, several projects based on low-cost environmental noise sensor networks have been developed [19,20,21,22].

These solutions show different approaches for implementing noise monitoring systems in smart acoustic sensor networks. In each reference, we can see that different open topics in noise measurement are covered. However, there are several common elements. These solutions use commercial hardware to produce a test concept of the proposed architecture. Not all references use exhaustive tests to measure quality characterization, and when these tests are implemented, they are only implemented from an acoustic point of view; no electrical tests are used, as suggested by the standard IEC 61672-3. In addition, these solutions measure only a few acoustic parameters, mainly the equivalent continuous sound level (Leq). Although some of these solutions point toward future trends in the application of algorithms for source identification, the equipment used is mono-channel, which reduces the possibility of implementing algorithms for source localization, one of the open topics for future noise measurement in cities.

To address these points, in this paper, a low-cost instrument to measure outdoor noise is presented. The equipment has been designed to form an electronics–microphone set, which allows it to be seen and used as a sensor device to be connected to microprocessor systems, thereby increasing its versatility and ease of use. The electronic device has been designed keeping in mind some of the challenges to be covered by future environmental noise monitoring networks. For this reason, the equipment incorporates two measurement channels, which together with a digital signal processor (DSP) will allow the future implementation of algorithms for detecting and locating noise sources. Likewise, noise measurement algorithms have been designed to meet the measurement requirements of type 1 instruments, so in the future, different types of microphones can be connected, thereby covering the different degrees of precision needed. 

The equipment has been designed with fully digital implementation capability to increase its quality and reliability and to reduce its cost. The system has been designed around a Texas Instruments C5000 DSP, due to its low power consumption and high performance. Our instrument implements two fully functional measuring channels, having the basic functionalities of a sound level meter together with the ability to perform octave-level and one-third octave-level frequency analyses.

One of the key elements in sound level meters is a condenser microphone, which has exceptional characteristics for measuring noise, such as a flat frequency response, a large dynamic range, high precision, and repetitiveness [23]. However, these characteristics make this type of instrument more expensive, hindering the widespread use of this type of microphone in the implementation of monitoring networks with a large number of nodes in smart cities. 

One of the open challenges in environmental noise sensor networks is to build mixed networks where low-cost instruments can be used without the overall quality of the network measures being substantially affected. To comply with these requirements, the use of condenser microphones must be heavily restricted. However, the equipment used must be subjected to the tests indicated by the standard for sound level meters to assure the quality of their measurements [12,13,14,15,16,17]. For this reason, and to characterize the quality and usability of the equipment, a set of exhaustive tests have been performed. These tests were mostly laboratory tests similar to those applied to sound level meters, including electric and acoustic tests [14,17]. One of the main objectives of the designed equipment is its capability to be used as a sensor in any acoustic sensor network. For this reason, the device has a simple interface for connection to microprocessor systems. The designed equipment was connected to a NRG2 panStamp wireless module. This module is based on the CC430F5137 system-on-chip (SoC) design, which provides a communication radio channel at free industrial, scientific and medical (ISM) bands (868 MHz). With this configuration, a set of eight instruments were built to be connected to an existing acoustic sensor network in the city of Málaga in Spain. Málaga is a city that is very concerned about the quality of life of its citizens [24] and has the infrastructure to carry out an outdoors test with the units we built.

## 2. System Description

**Figure 1:**
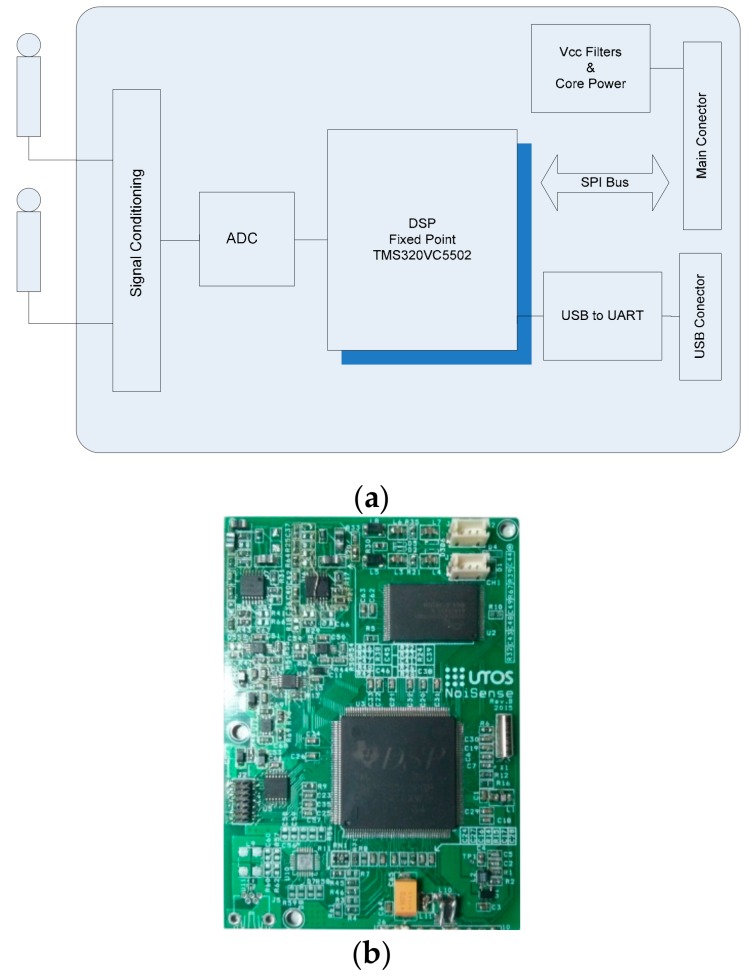
(**a**) Block diagram of the original equipment manufacturer (OEM) module for noise measurement; (**b**) final circuit implementation of 87 × 62 mm. DSP = digital signal processor; SPI bus = serial peripheral interface; ADC = analog to digital converter; UART = universal asynchronous receiver-transmitter.

Figure 1 shows a block diagram of the designed instrument and the form factor of the implemented module. The set formed by the microphone and the designed card was implemented as an original equipment manufacturer (OEM) module, allowing it to be easily integrated into any monitoring network. The core of the system is a digital signal processor that digitally implements all acoustic functions in order to achieve a more robust, economical, and adjustment-free architecture. Power consumption is a very important issue in systems designed for monitoring purposes. Usually, these systems are installed in remote or isolated places and are powered by solar panels. Thus, a family of fixed-point and low power consumption DSPs has been chosen. The use of a DSP allows us to implement many parameters without an increase in hardware cost, requiring only firmware upgrades. This system is capable of measuring, in real time, the following parameters for both channels simultaneously: the equivalent continuous sound level (Leq) (with Z, C, and A frequency weightings); the peak level (with Z, C, and A frequency weightings); the maximum and minimum levels with (Z, C, and A frequency weightings); the impulse, fast, and slow time weightings; seven percentiles (1%, 5%, 10%, 50%, 90%, 95%, and 99%); as well as continuous equivalent sound pressure levels in the one-third octave and octave frequency bands. 

### 2.1. Hardware Solution

**Figure 2:**
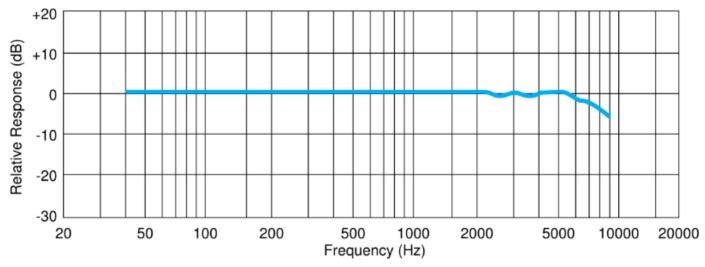
Panasonic WM 63-PR microphone frequency response.

One of the most import elements in a noise measurement system is the microphone, which determines the overall quality of the equipment. In order to build cost-effective solutions, condenser microphones must be avoided due to their high price. The microphone used is an electret Panasonic WM 63-PR [25], whose frequency response is shown in Figure 2. The frequency response and dynamic range of the microphone suggest that it can be used to design an instrument with characteristics similar to type 2 sound level meters, according to the IEC 61672 standard [13], with a frequency range of 63 HZ to 8 kHz. 

**Figure 3:**
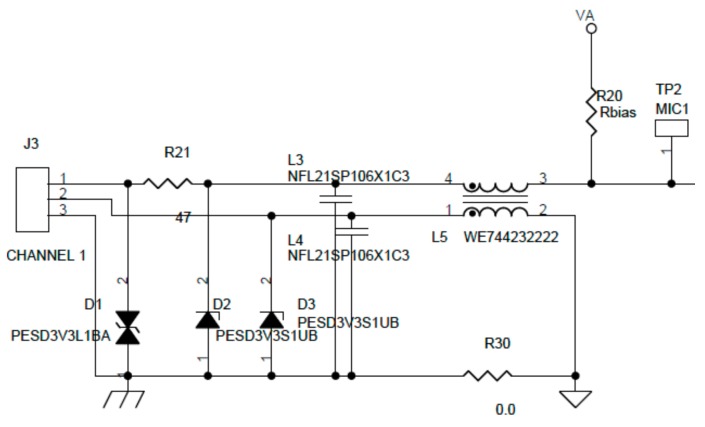
Filter stage for noise minimization and electrostatic discharge (ESD) and electromagnetic interference (EMI) protection.

The first block in Figure 1a shows the signal conditioning stage, which is used to adapt the signal from the microphone to the analog to digital converter (ADC). This stage is formed by two active filters. One of them is a high-pass filter with a cutoff frequency of 5 Hz, and the other is a low-pass filter with a cutoff frequency of 22.7 kHz; both feature a unity gain. To adapt the microphone’s signal level to the dynamic input range of the ADC, a gain stage was added. To minimize the noise and protect the equipment against electrostatic discharge (ESD) and electromagnetic interference (EMI), a front end circuit was added between the microphone and the first filter. Figure 3 shows this circuit.

**Figure 4:**
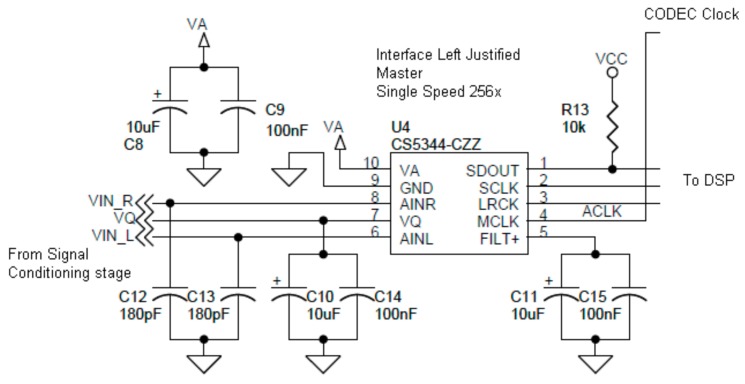
ADC connection.

The ADC used is a CS5344 from Cirrus Logic [26], whose main features are a 24-bit sigma–delta converter, a sample rate up to 108 kHz, a dynamic range of 98 dB at 5V, a power consumption less than 40 mW at 3.3V, and a single power supply. The ADC is connected to the DSP using an multichannel buffered serial port (MCBSP) interface and left-justified with 256x speed. The transfers are managed using a direct memory access (DMA) channel; the block transfer includes 2048 samples for the two channels. Figure 4 shows the ADC connection. 

The core of the system is a Texas Instruments fixed-point DSP (TMS320VC5502) in a low-profile quad flat package (LQFP) [27]. To optimize the system, all algorithms are executed in the internal RAM of the DSP, so only flash memory is connected to the DSP itself, which is used to store the code with the algorithms. This code is downloaded to the RAM at boot time. All algorithms are implemented through cycle optimization and have been coded in assembly language to reduce the code size and improve run time. In this way, the code can be allocated in the internal RAM of the DSP, and a lower main frequency can be used for the DSP, thereby reducing the overall power consumption [28]. 

The system is designed to be used similarly to an OEM module to measure environmental noise; it can be managed by an external control unit using a serial peripheral interface (SPI) interface. This equipment can be connected to a microprocessor system to manage it using a simple interface. In this interface, there are four SPI signals, four control lines (these lines are used for the microprocessor system to reset the instrument, to send a configuration command, to start the measurements, and for when the equipment sends data to the microprocessor), and two lines for the power supply.

### 2.2. Software Implementation

**Figure 5:**
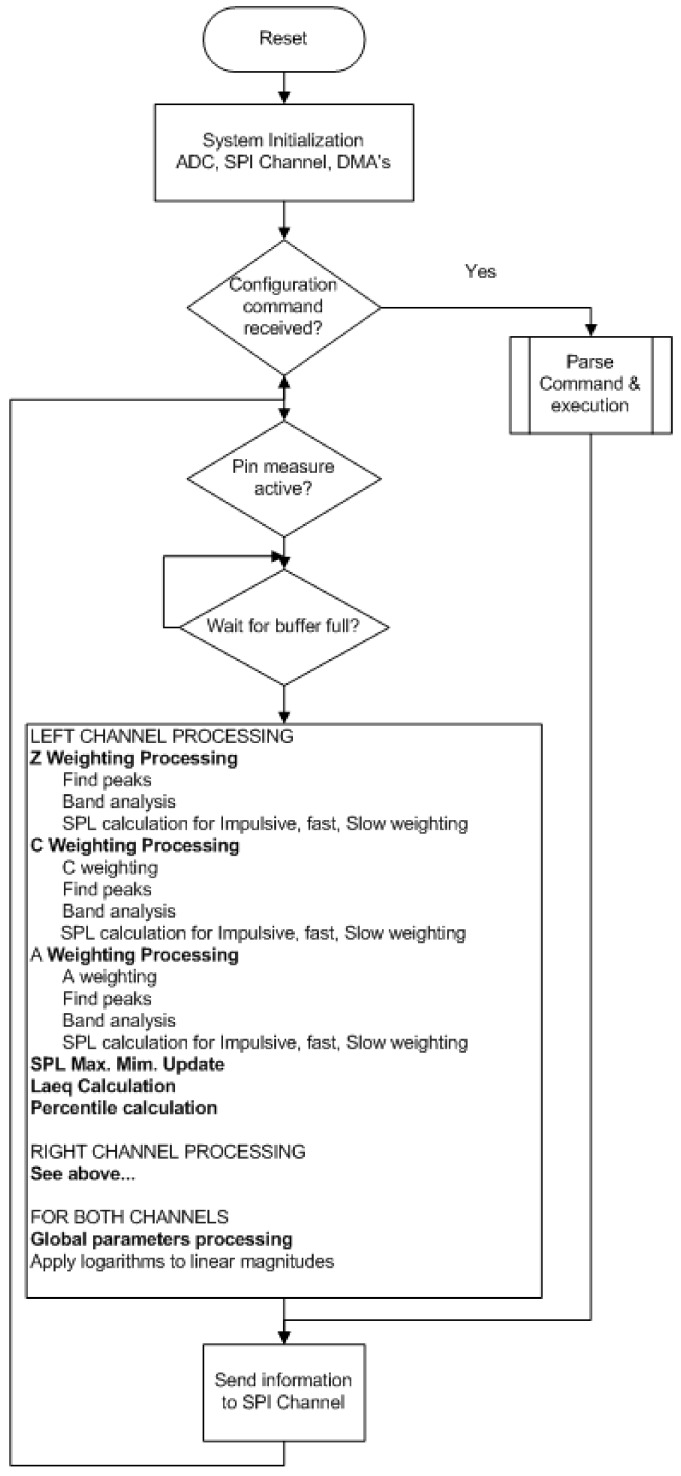
Software flowchart.

Software for non-time-critical tasks, such as system initialization, commands parsing, and flow control, were implemented in the C language. However, the audio signal processing was coded in assembly language to improve its run time. Figure 5 shows the main flowchart of the code.

For the time weighting processing (impulsive, fast, and slow), the following formula is used:

(1) $$L_{m}\left[n\right]=\frac{1}{Fs\cdot T_{m}}\left[\left(Fs\cdot T_{m}-1\right)L_{m}\left[n-1\right]+x^{2}\left[n\right]\right]$$

where T_m_ is 0.125 seconds for fast weighting, 1 second for slow weighting, and 0.035 seconds for impulse weighting [12]. As divisions are computationally expensive for fixed point DSPs, the values (Fs·Tm – 1) and 1/(Fs·Tm) are pre-calculated to optimize run time. In the few places where a division cannot be pre-computed, the Newton–Raphson method is used to quickly obtain the reciprocal and perform a multiplication instead of a division. The impulse time weighting detector also requires an additional filter at the output, with a maximum drop slope of 2.9 dB.

The frequency weighting filters A and C were calculated using MATLAB [29]. First, the analog filters have been designed using the poles shown in Table 1 [12]. Then, the analog filters were transformed into digital filters using the impulse invariance method [30].

**Figure 6:**
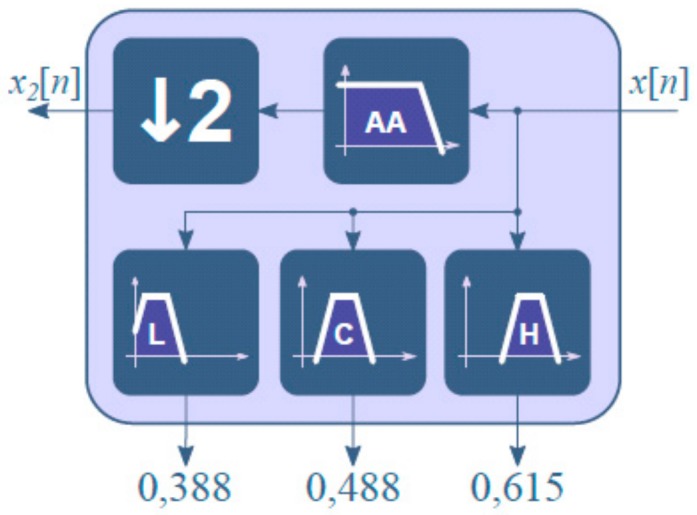
Base block used to build the bank filter. Frequencies are normalized to sampling frequency (Fs/2).

The IEC 61260-1 standard allows two methods for design of the octave and one-third octave filters in base 10 (preferred) and in base 2 (allowed) modes [15]. For optimization and computational efficiency, the filters were implemented in base 2. A multirate filter structure was used for the design of the third octave filter bank. This allowed us to simplify the design process; instead of designing 30 filters, it was only necessary to design the three corresponding to the octave of greatest frequency, with a central frequency of 16 kHz, in addition to the anti-aliasing filter for the decimator. Figure 6 shows the base block used to build the bank filter. This block consists of a down sampler (by 2), an anti-aliasing filter, and three filters with normalized center frequencies of 0.388, 0.488, and 0.615 [31].

**Figure 7:**
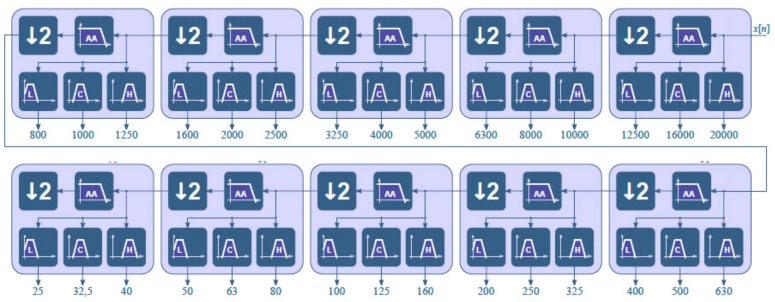
Filter bank for the one-third octave. The frequencies are shown in Hz.

The final frequencies obtained in the real domain have slight differences compared to the nominal values. However, this situation is understood and allowed in the IEC 61260 standards [15,16,17]. Figure 7 shows the complete filter bank that was implemented. This kind of implementation reduces the workload because only four filters are designed. This methodology also has two additional advantages: avoiding the cutoff frequencies to get close to the limit frequency’s minimum and maximum (0 and 32,768 Hz), and reducing the processing power required by the DSP. Due to the successive decimation performed at each stage, the processing load required to compute the whole filter bank is about twice that required to calculate the basic building block shown in Figure 6.

To obtain the response of the octave band filter bank from the one-third octave band filter bank, it is sufficient to add (in linear magnitude, not in dB) the values obtained from the three adjacent one-third octave filters in the octave of interest.

## 3. Implemented System Verification

In order to verify the designed system, the equipment underwent different tests to analyze the acoustic and electrical properties. The electrical tests were used to verify the quality of the digital implementation (digital filters, algorithms, etc.) and the analog input chain, while the acoustic tests were used to verify the microphone’s behavior. Both tests together were used to verify the overall features of the equipment.

### 3.1. Electrical Test

The third section of the international standard IEC 61672 specifies the tests required to verify the frequency weightings implemented in a sound level meter [14], which should be determined relative to the response at 1 kHz using steady sinusoidal electrical input signals. At the reference level range, and for each frequency, the 1 kHz input signal should be adjusted to yield an indication that is 45 dB less than the upper limit for the sound level meter [14]. The tolerance limits are specified in IEC 61672-1 [12]. For class 1 SLM, the test should be performed with nine frequencies at nominal octave intervals from 63 Hz to 16 kHz. For a class 2 SLM, eight frequencies should be used from 63 Hz to 8 kHz. Table 2 shows the results obtained. Here, the equipment fully fits the requirements of a class 1 SLM.

For the level linearity test, the IEC 61672-3 states that the test must be performed using a sinusoidal electrical signal at a frequency of 8 kHz that varies its amplitude for the SLM linear measurement range [14]. The linearity will be measured in steps of 5 dB until it reaches 5 dB before the extreme limits of the linear range. Then, the steps will be 1 dB increments until the limits are reached. Table 3 shows the results obtained for the linearity test. This table only shows the central values and extremes for clarity. With these results, we can establish the linear dynamic range of the equipment in 80 dB. 

The instrumentation equipment used for the electrical tests included:

- Multifunction acoustic calibrator Brüel & Kjaer B&K 4226;
- Signal Generator Stanford DS360;
- Multimeter Keithley 2015-P.

The reference conditions were Temperature = 23 °C ± 2 °C; Relative humidity = 50% ± 20%; Atmospheric pressure = 95 kPa ± 10 Pa.

### 3.2. Acoustic Test

**Figure 8:**
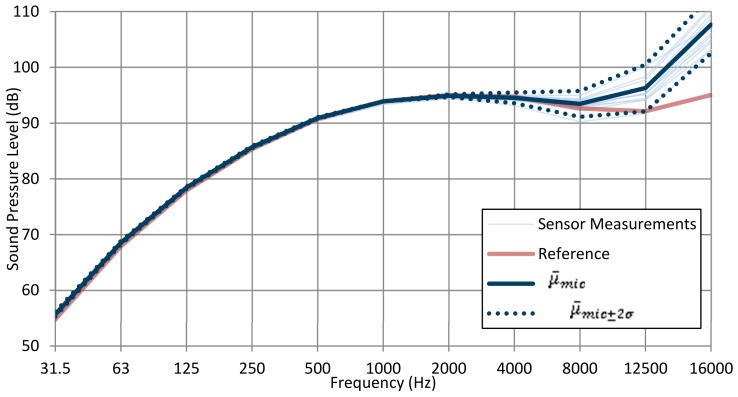
Tests results using a multifrequency calibrator.

A set of tests was performed using a multifunction acoustic calibrator (B&K 4226) in a calibration laboratory. The use of a multifrequency calibrator allowed us to determine the microphone’s influence on the equipment, thus removing the effects of diffraction and refraction that appear when the microphone is inside an acoustic field. Thirteen different devices were tested, and Figure 8 shows the results obtained. The individual measurements of each of the devices are shown in light blue, and the measurement corresponding to a type 1 reference sound level meter is shown in gray, adjusted to the level offered by the 1 kHz reference microphone. Also, the mean (${\bar{\mu }}_{mic}$) and the typical deviation with a 95% confidence interval (${\bar{\mu }}_{mic}\pm 2\sigma$) are shown in solid and dotted dark blue, respectively.

The variability of the thirteen units with their microphones is low and constant for frequencies between 31.5 Hz and 4000 Hz (the predominant bandwidth in city noise). This deviation increases for higher frequencies. Table 4 shows the values of the typical deviation.

**Figure 9:**
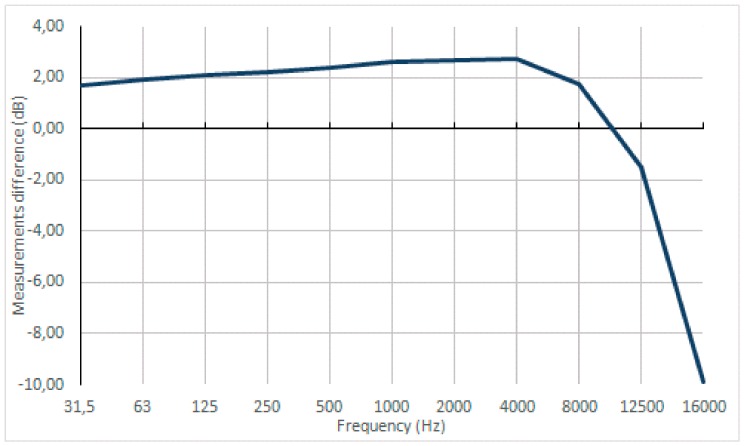
Difference in the average value between the type I reference equipment and the mean of the measures of the equipment designed for each frequency band.

Figure 9 shows the difference between the mean of the thirteen units and the measures of the reference equipment (type 1). This difference is less than 3 dB at all frequencies, except at 16,000 Hz, where the difference is 10 dB.

**Figure 10:**
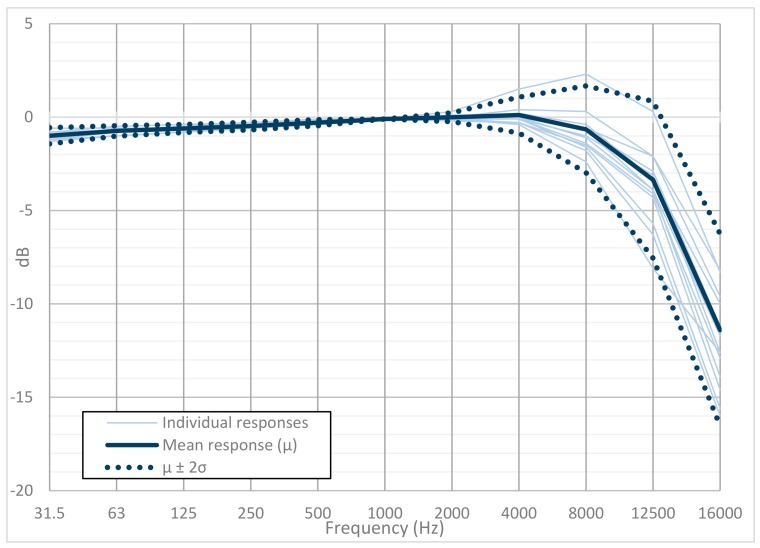
Frequency response for the thirteen units, including the mean and standard deviation for 2 σ.

Figure 10 shows the response of the designed equipment in reference to a tone of 1 kHz and a 94 dB sound pressure level, the reference commonly used in the characterization of this type of measurement instrument. At 1 kHz, all frequency responses are about 0 dB, and the variability is visible with respect to the other frequency bands. For this equipment, the variation of the response is reduced (almost flat) to 4 kHz, thereby worsening its frequency response noticeably from 8 kHz.

Finally, the closest equipment to the average was chosen to be tested according to the IEC-61672-3 standard [14]. Table 5 shows the measurements and their comparisons with the requirements specified in the aforementioned standard. At 8 kHz, the equipment does not meet the specifications of a type 1 instrument. The values here fully fit with those of type 2 equipment [12]. 

### 3.3. System Verification Conclusions

Once the different tests had been carried out, we concluded that from the perspective of digital signal processing, this equipment is capable of complying with the specifications of type 1 instruments up to 16 kHz. However, the use of a low-cost electret microphone means that the acoustic behavior can only fully fit a type II instrument when tests are done using a multifrequency calibrator for a frequency range up to 8 kHz. 

## 4. Results: Deployment in Málaga City

**Figure 11:**
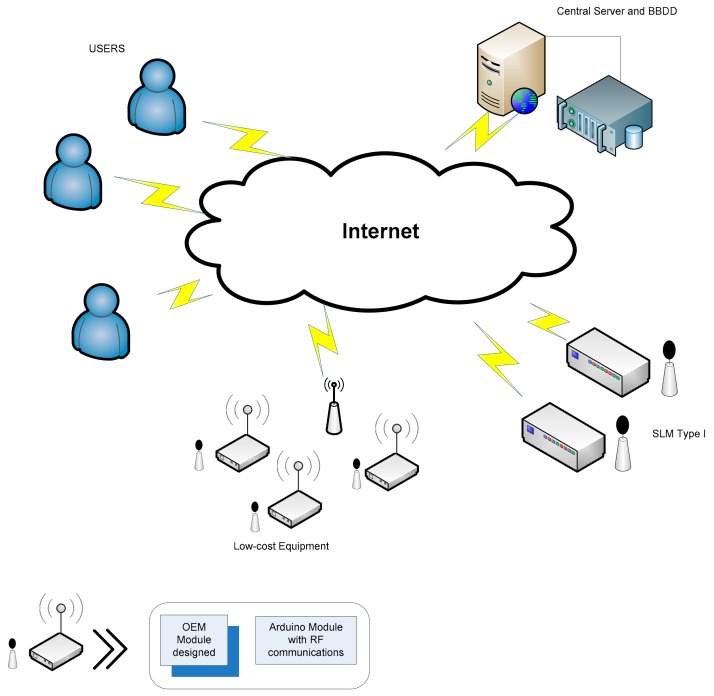
Network architecture and details of the low-cost monitors.

Once it was verified that the equipment offers sufficient quality to measure the noise in cities, the next step was to test the OEM module design in a real environment. The Spanish city of Málaga had optimal conditions to carry out this experiment. Málaga is a modern city concerned about the problem of outdoor noise; it has an operational data monitoring platform with the ability to insert new nodes, and it is possible to access these data to analyze them. For these reasons, eight units were deployed in the city to verify their behavior under real operating conditions over three months. The noise monitoring systems were located on the façades of residential buildings, in areas affected by night-time leisure noise located on pedestrian streets, and near bars, pubs, restaurants, and terraces [32]. To help to verify the quality of the measurements of these units, the equipment was installed in places where type 1 monitors were available. To keep the power consumption as low as possible, the equipment under study was configured to provide data every minute to reduce the power used for communication. In this way, the equipment used batteries, making the installation process easier. Unlike the network of type I monitors, where the equipment sends data directly to the central server through a general packet radio service (GPRS) connection, the terminals under study send their data via radio (868 MHz) to several access points, which then send the data to the same central server through a GPRS connection. Figure 11 shows the architecture used. The implemented OEM module was connected to a NRG2 panStamp-based CPU, and the code was developed using Arduino. This CPU obtained data from the OEM module and sent it by radio to the access point, which transformed it into frames for the existing architecture. 

In addition, to verify the evolution of the equipment, three tests measurements were carried out in situ to determine the evolution of the operation of the units. One was performed after the outdoor installation using an acoustic calibrator (94 db). Table 6 shows the results for the eight devices and their locations.

The second verification was carried out a month later; in this case, a type 1 instrument was placed next to the microphone of the equipment under study to measure the same noise at the same time. A three-step cycle of about ten minutes was performed. First, the background noise was measured for three minutes. Then, a five-minute stationary measurement was taken using a speaker placed above the vertical axis of the microphones with white noise. Then, the background noise was measured again for three minutes. Finally, at the end of the measurement period, another comparison was made using an acoustic calibrator for the eight devices used. After analyzing the data from these three tests, two of the units presented important errors, so they were removed for the final analysis under suspicion that they had been damaged during the measurement period.

**Figure 12:**
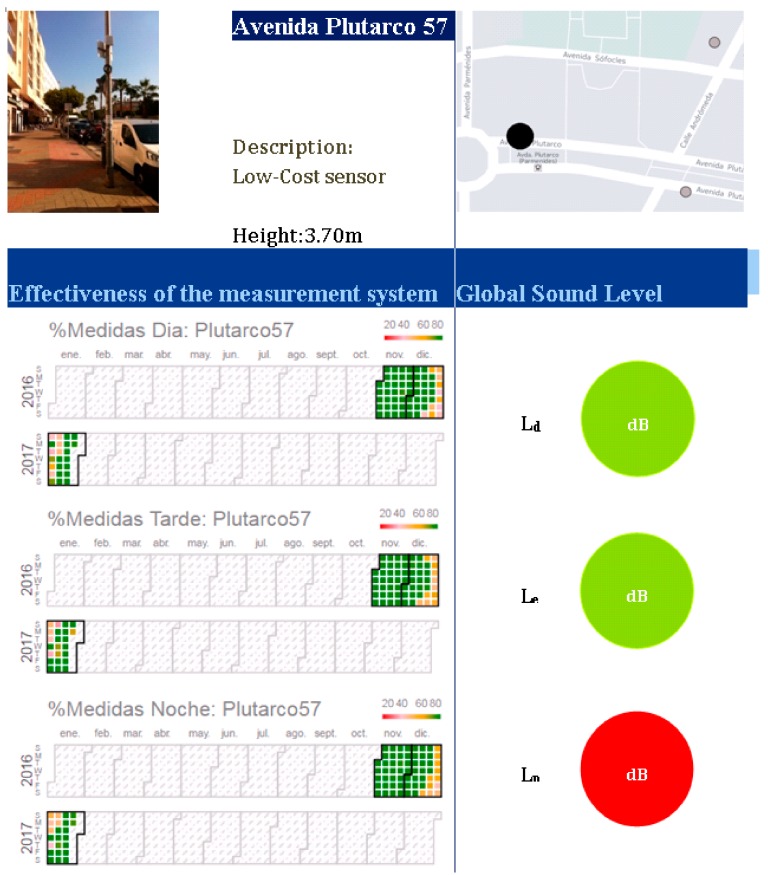
Information template used for each measurement point.

The data from the central server can be remotely consulted and downloaded using a web browser. After three months of the campaign, an exhaustive analysis of the data stored on the central server was performed. The downloaded data were analyzed and a report was made. Figure 12 shows the information for each monitor. The top part shows a view of the equipment installation, along with its localization on the city map. The equivalent noise levels for the day (Ld), evening (Le), and night (Ln) were then calculated. Using a semaphore color code, the obtained noise levels were classified. A figure with a calendar shows the percentage of measurements received for each day. Days that received less than 30% data were deleted for the calculation.

**Figure 13:**
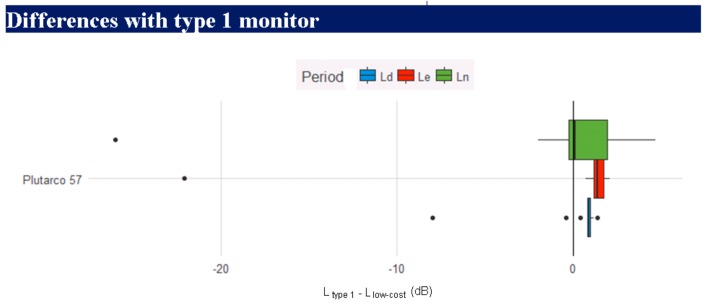
Box plot diagram to compare the measurements between instruments.

The report also shows box plot figures with the measurement difference between a type 1 instrument and a low-cost instrument. Figure 13 shows a diagram for the equipment placed on Plutarco Street. We can see that there is a greater dispersion in sound levels at night, which is due to the minimum value of the dynamic input range of the instrument. The figure also shows the outlier values, where one can see the low outlier numbers over three months.

Table 7 shows the average values (day, afternoon, and night) measured during the 84 campaign days for type 1 and low-cost instruments.

The device installed on Plutarco Street 57 shows a great difference in the night’s equivalent noise level. After a deeper analysis of the data, this deviation was found to be due to the measurements made during the Christmas season. The Christmas lights installed near the devices produced some interference in the measurements.

## 5. Discussion

When the design started, the main objective was to obtain a noise measurement device with the following features:

- Quality and characteristics comparable to the sound level meters that are used today to measure this type of magnitude, according to the standards and regulations that apply to them;
- Low cost required to build a large-scale monitoring network;
- Processing capabilities for the detection of target sources and their spatial localization;
- Easy integration into sensor networks.

All these objectives, also mentioned by other authors, have been covered [19], and the developed equipment was conceived as a generic OEM card. The unit can be controlled by two serial interfaces: SPI or USB. These interfaces are widely used in microprocessor-based systems, which allows this device to be easily integrated, and thus can be used to build noise measurement equipment in a simple way. The developed equipment has the ability to measure two channels simultaneously and implement the basic functions of an integrating sound level meter, carrying out measurements simultaneously for A, C, fast, slow, and impulsive weights. It also allows one to obtain the maximum and minimum values, perform an analysis in the octave and one-third octave frequency bands, and calculate the percentiles of the measurements, resulting in eighty-six parameters being measured for each channel in real time. All these parameters are calculated digitally; the code optimization allows the DSP to compute all these parameters for both channels in real time using a clock frequency of 60 MHz. As the DSP allows one to raise the clock speed to 300 MHz, further algorithms for tasks such as noise source identification and pattern recognition of acoustic footprints can be implemented in the future using this equipment after a software upgrade. 

The electrical tests applied to the equipment show that the electronics and algorithms implemented have the behavior of a type 1 sound level meter for an input frequency range spanning from 10 Hz to 20 KHz and a linear dynamic range of 80 dB. However, the use of an electret microphone instead of a condenser microphone reduces the electric performance of the equipment, limiting its characteristics to those of a type 1 instrument for a frequency range up to 8 kHz (Table 5). For type 2 instruments, the tolerance allowed at 8 kHz increases to ± 5.6 dB. Thus, the equipment complies with the requirements of a type 2 instrument according to IEC61672-3 [14]. The use of the electret microphone also reduces the acoustic features of the equipment. However, this error can be assumed for the application of acoustic noise measurement in cities, as has been shown with the correlation analysis using the measurements of type 1 equipment installed as a reference in the measurement sites.

Eight units were deployed in the city of Málaga from November to January to verify their operation under real conditions of outdoor noise measurement. Some very interesting conclusions were obtained when analyzing the data uploaded by the units compared to the type 1 equipment used to contrast the results. Finally, only six devices could be used for the final results, since two of them were damaged and suffered large deviations in their measurements after the three months of the measurement campaign. The results obtained for four of the devices were very good; however, two of them presented a deviation in their data right at the limit of what is acceptable for a measurement, with quality similar to that provided by type 2 instruments. A detailed study of these data showed that the devices were more influenced by meteorological phenomena, such as rain, than type 1 monitors. In addition, it was possible to verify how—coinciding with the installation and lighting of the streets for Christmas—some measurements were affected by electromagnetic interference. 

To summarize, the designed equipment can easily be integrated by third party users into any wireless sensor network due to the available digital interface. The device is designed to have SLM functionality and is capable of measuring 86 parameters by channel, including frequency analysis. This creates new possibilities for low-cost sensor networks, where traditionally only the equivalent continuous sound level (Leq) is measured. The designed instrument had an input linear range of 80 dB and passed the electrical test according to the standards applied to a type 1 SLM, although the use of an electret microphone reduced the quality of the measurements to those indicated for type 2 instruments. However, this is an important point. If the device was to be built with a better microphone (at present, microelectromechanical (MEMS) microphones possess good features similar to those of traditional condenser microphones [19,32]), the instrument could be a very good solution for creating low-cost sensor networks with high measurement quality.

The results have shown that there are two aspects to need to be improved in the proposed solution. One is the vulnerability that some units showed to electromagnetic interference. The case used to house the equipment was a standard plastic case. To improve this feature, a metal case or plastic cases with anti-EMI treatments must be used. The other major change would be the use of new technological advances in MEMS microphones to achieve better measurement quality. Thus, the next step will be to modify the signal conditioning stage to use MEMS microphones [33].

## Figures and Tables

**Table 1:** Poles used to design the analog filters.

Weighting	Poles
C	Two real poles at 20.6 Hz Two real poles at 12,200 Hz
A	Poles in C and One pole at 107.7 Hz One pole at 737.9 Hz

**Table 2:** Results obtained for the frequency weighting test.

	Frequency (Hz)	Frequency Weights (dB)	Correction (dB)	Read Level (dB)	Expected Level (dB)	Deviation (dB)	U(uncertainty) (dB)		Positive Tolerance (dB)	Negative Tolerance (db)
101.20	63	−26.2	0	75.2	75.0	0.20	0.18	1.5		−1.5
91.10	125	−16.1	0	75.1	75.0	0.10	0.18	1.5		−1.5
83.60	250	−8.6	0	74.9	75.0	−0.10	0.18	1.4		−1.4
78.20	500	−3.2	0	75.0	75.0	0.00	0.18	1.4		−1.4
75.00	1000	0	0	75.0	-	-	-	-		-
73.80	2000	1.2	0	75.0	75.0	0.00	0.18	1.6		−1.6
74.00	4000	1	0	74.9	75.0	−0.10	0.18	1.6		−1.6
76.10	8000	−1.1	0	75.0	75.0	0.00	0.18	2.1		−3.1
86.10	16,000	−6.6	0	75.2	75.0	0.26	0.18	3.5		−17

**Table 3:** The results obtained for the linearity test. Values marked with “…” are omitted for clarity (the device passed the test). SLM = sound level meter.

Applied SPL (dB)	Frequency (Hz)	SLM Level (dB)	Expected Level (dB)	Deviation (dB)	U (dB)	Positive Tolerance (dB)	Negative Tolerance (db)	Test Result
122.1	8000	118.9	122.0	−2.1	0.14	1.1	−1.1	ERROR
121.1	8000	118.9	121.0	−1.1	0.14	1.1	−1.1	ERROR
120.1	8000	118.5	119.0	−0.5	0.14	1.1	−1.1	PASS
…	…	…	…	…	…	…	…	…
105.1	8000	104.0	104.0	0.0	0.14	1.1	−1.1	PASS
100.1	8000	99.0	99.0	0.0	0.14	1.1	−1.1	PASS
95.1	8000	94.0	-	-	-	-	-	
85.1	8000	84.0	84.0	0.0	0.14	1.1	−1.1	PASS
80.1	8000	79.0	79.0	0.0	0.14	1.1	−1.1	PASS
…	…	…	…	…	…	…	…	…
39.1	8000	38.9	38.0	0.9	0.14	1.1	−1.1	PASS
38.1	8000	38.1	37.0	1.1	0.14	1.1	−1.1	ERROR
37.1	8000	37.4	36.0	1.4	0.14	1.1	−1.1	ERROR

**Table 4:** Standard Deviation for the frequency bands.

Frequency (Hz)	31.5	63	125	250	500	1000	2000	4000	8000	12,500	16,000
Standard deviation (dB)	1.3	1.3	1.3	1.3	1.3	1.3	1.3	1.4	1.8	2.4	2.6

**Table 5:** The obtained results. At 8000 Hz, we can see that the microphone response deviation falls outside the positive tolerance for a type I instrument (marked in grey).

Applied SPL (dB)	Frequency (Hz)	Frequency Weights (dB)	Correction (dB)	Read Level (dB)	Expected Level (dB)	Deviation (dB)	U (dB)	Tolerance (dB)
93.96	63	−26.2	0.0	66.45	65.90	0.55	0.23	1.5; −1.5
93.95	125	−16.1	0.0	76.40	75.99	0.41	0.20	1.5; −1.5
93.95	250	−8.6	0.0	83.70	83.49	0.21	0.20	1.4; −1.4
93.94	500	−3.2	0.0	88.95	88.88	0.07	0.23	1.4; −1.4
93.96	1000	0	0.1	92.00	--	--	--	--; -
93.97	2000	1.2	0.4	92.80	92.91	−0.11	0.20	1.6; −1.6
93.96	4000	1	1.6	92.35	91.50	0.85	0.23	1.6; −1.6
93.90	8000	−1.1	2.9	91.05	88.04	3.01	0.24	2.1; −3.1

**Table 6:** Verification values after installation outdoors (94 dB applied).

Monitor Location street	Value (dB) at Calibration	Monitor Location street	Value (dB) at Calibration
Andromeda, 9	93.6	Plutarco, 20	93.2
Angel	93.7	Plutarco, 57	93.8
Capitán	93.6	Velázquez	93.4
M. Vado Maestre, 4	93.6	M. Vado Maestre, 6	93.4

**Table 7:** Ld, Le, and Ln levels. The left values are from low-cost monitors and the right values are from the type 1 monitors used as a reference.

Monitor’s Location	L_d_ (dB)	L_e_ (dB)	L_n_ (dB)
Andromeda, 9	62/63	62/63	57/59
Angel	65/67	69/71	71/70
Capitán	69/74	78/79	74/76
M. Vado Maestre, 4	65/67	66/71	68/71
Plutarco, 20	61/63	62/64	58/59
Plutarco, 57	65/65	67/64	71/62
